# Experimental determination of translational starts using peptide mass mapping and tandem mass spectrometry within the proteome of *Mycobacterium tuberculosis*

**DOI:** 10.1099/mic.0.2006/001537-0

**Published:** 2007-02

**Authors:** Stuart C. G. Rison, Jens Mattow, Peter R. Jungblut, Neil G. Stoker

**Affiliations:** 1 The Royal Veterinary College, Royal College Street, London NW1 0TU, UK; 2 Max-Planck-Institute for Infection Biology, Core Facility Protein Analysis, Campus Charité Mitte, Schumannstr. 21/22, D-10117 Berlin, Germany

## Abstract

Identification of protein translation start sites is largely a bioinformatics exercise, with relatively few confirmed by N-terminal sequencing. Translation start site determination is critical for defining both the protein sequence and the upstream DNA which may contain regulatory motifs. It is demonstrated here that translation start sites can be determined during routine protein identification, using MALDI-MS and MS/MS data to select the correct N-terminal sequence from a list of alternatives generated *in silico*. Applying the method to 13 proteins from *Mycobacterium tuberculosis*, 11 predicted translational start sites were confirmed, and two reassigned. The authors suggest that these data (be they confirmation or reassignments) are important for the annotation of both this genome and those of organisms with related genes. It was also shown that N-acetylation, reported to be rare in prokaryotes, was present in three of the 13 proteins (23 %), suggesting that in the mycobacteria this modification may be common, and an important regulator of protein function, although more proteins need to be analysed. This method can be performed with little or no additional experimental work during proteomics investigations.

## INTRODUCTION

The sequencing of complete genomes allows every potentially encoded protein to be identified. In prokaryotes, gene finding using a combination of bioinformatic factors (homology with other predicted proteins, third base preference, etc.) is quite efficient, and is generally achieved by a combination of automated and manual curation (Brent, 2005). However, the final proof that a gene is expressed as a protein can only be provided by experimental protein analysis. Proteomic approaches have been used to confirm many predicted genes, as well as to identify genes not easily found bioinformatically (Jungblut *et al.*, 2001). However, there is one aspect of the annotation that is difficult to predict and usually remains experimentally untested: the translational start site (TSS).

Determining the TSS accurately is important, not only because this defines the amino acid sequence of the protein (as the stop codon is unambiguous), but also because this defines the upstream region in the DNA. Genome-wide and focused studies of promoter structure and regulatory motifs depend on the intergenic regions defined by the gene-finding process (Edwards *et al.*, 2005; Salgado *et al.*, 2000). The TSS assignment therefore affects the analysis of both protein function and transcriptional regulation.

Traditionally, TSS identification has been achieved using N-terminal sequencing by Edman degradation (Edman, 1950). This is often technically demanding, and requires large (picomole) quantities of protein. Furthermore, some proteins are blocked through N-terminal modifications and cannot be sequenced. Proteomic approaches using sensitive MS methods (Jungblut *et al.*, 1999, 2000) have revolutionized protein analysis because of the speed and sensitivity of the technology, but are not used to identify TSSs.

In this paper we apply rapid proteomic analysis methods to the issue of identifying TSSs. As the same data generated for protein identification could simultaneously identify protein starts, this is therefore a highly efficient approach. We were able to confirm the predicted N termini of 11 proteins, and to correct the predictions for two proteins from *Mycobacterium tuberculosis*. We also demonstrated that in contrast to the predicted situation in *Escherichia coli*, a high proportion (23 %) of the proteins showed N-terminal acetylation.

## METHODS

### Alternative start codon identification.

**Fig. 1. f1:**
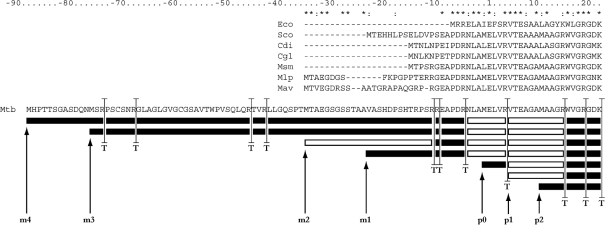
General strategy for identifying alternative translation start positions and tryptic digest peptides: application to GlpX (Rv1099c). The six alternative translation starts for GlpX are indicated by the labelled arrows. The minus (m)1, m2, m3 and m4 starts are located upstream of the original (p0) translation start indicated in the *M. tuberculosis* genome annotation. The plus (p)1 and p2 starts are downstream of the original translation start prediction. The putative trypsin cleavage sites are indicated by the ‘T’-labelled double-tailed bars. The resulting tryptic peptides are shown by the horizontal boxes. Tryptic fragments identified by MS/MS are shown in white; those not detected are shown in black. The figure shows that the detected fragments could only have resulted from the digest of the m2 variant. For reference, the *M. tuberculosis* sequence was aligned with the N-terminal region of six other actinomycete GlpX homologues, as well as with the *E. coli* homologue. Asterisks indicate perfectly conserved residues; colons indicate conserved substitutions (start codon methionines are ignored). The *C. glutamicum* homologue TSS (at position −14) has been confirmed experimentally (Rittmann *et al.*, 2003). Organisms are *E. coli* (Eco), *Streptomyces coelicolor* (Sco), *Corynebacterium diptheriae* (Cdi), *C. glutamicum* (Cgl), *Mycobacterium smegmatis* (Msm), *Mycobacterium leprae* (Mlp), *Mycobacterium avium* (Mav) and *M. tuberculosis* (Mtb).

The following strategy was used to generate alternative TSSs for each predicted gene in the *M. tuberculosis* genome. Each protein coding sequence in the EMBL entry [identified as a coding sequence (CDS) in the feature table] was considered in turn. The region upstream of the gene was scanned until an in-frame stop codon was identified; by definition, alternative start codons for the gene cannot be found upstream of such a stop codon. The in-frame codons downstream of this stop codon were scanned, and the position and triplet code of each alternative start codon (ATG, GTG or TTG) were recorded (these are the ‘m’ start codons in Fig. 1). Similarly, in-frame codons downstream of the original start codon (p0) were scanned for alternative start codons. Only the first two downstream alternative start codons were considered at this stage (these are the p1 and p2 codons in Fig. 1). For each predicted alternative start, the new protein sequence encoded was generated. Thus, from the 3999 coding sequences listed in AL123456.2, 15 199 alternative start protein sequences were identified. This procedure was performed using a Perl program (AlternaStart.pl). The output of program AlternaStart.pl is available as Supplementary Table S1. All sequences in this manuscript were derived from the *M. tuberculosis* H37Rv complete genome entry in the EMBL database [accession no. AL123456 (version 2)].

### *In silico* tryptic digestions.

The alternative protein sequences generated above were subjected to an *in silico* tryptic digest using the proteogest software from the University of Toronto (Cagney *et al.*, 2003) (downloaded from http://www.utoronto.ca/emililab/proteogestcode.htm). This run was performed using the standard tryptic digest settings (no missed cleavages, no modification, no cleavage where R or K are followed by P). The output of the proteogest program is available as Supplementary Table S2.

### Protein test set selection.

In order to assess the feasibility of our strategy, we decided to focus initially on the proteins most likely to have their definitive translation start identified by MS. Thus, the alternative protein sequences were screened according to a number of technical criteria. First we discarded any protein not in a set of 289 proteins for which a 2D-gel spot had been identified in a previous large-scale proteomics analysis of *M. tuberculosis* (http://web.mpiib-berlin.mpg.de/cgi-bin/pdbs/2d-page/extern/index.cgi). We next eliminated any protein for which the predicted mass of the first N-terminal tryptic fragment of the p0 protein was not between 800 and 2000 Da (the best resolution range for our MS equipment), and proteins without an arginine at the C-terminal end of the p0 tryptic peptide [as ionization in MALDI-MS is less effective in lysine-containing peptides than in arginine-containing ones (Krause *et al.*, 1999)]. The remaining 76 proteins were then ranked according to the minimal number of alternative starts (i.e. least total number of ‘m’ and ‘p’ variants) and least number of fragments assuming a p0 start. This selection and classification task was performed using the ParseProteogest.pl program, which also calculates N-terminal tryptic fragment weights, including possible modifications such as methionine formylation and fragment acetylation. The output of the ParseProteogest.pl program is available as Supplementary Table S3. The data for *M. tuberculosis* protein GlpX (Rv1099c) were added to the list, because although the protein does not meet all our selection criteria, we had strong previous evidence of incorrect translational start prediction for it (Movahedzadeh *et al.*, 2004). From these proteins, the 15 with the best available spectral data were selected for further investigation (Table 1). All these proteins were tested for the presence of a signal peptide using the online SignalP 3.0 resource available at http://www.cbs.dtu.dk/services/SignalP/ (Bendtsen *et al.*, 2004), and no signal peptide was detected in any of them.

### 2D gel electrophoresis (2-DE)/MALDI-MS and MS/MS.

Cellular proteins of *M. tuberculosis* H37Rv were prepared and 300 μg analysed by 2-DE, as previously described (Jungblut *et al.*, 1999). Protein spots on analytical gels were visualized by Coomassie brilliant blue G250 staining (Doherty *et al.*, 1998). Spots were excised and digested in-gel (Lamer & Jungblut, 2001). MALDI-MS was performed on a PerSeptive Voyager Elite time-of-flight instrument (PerSeptive), for which 0.5 μl peptide solution was mixed with an equal amount of dehydroxybenzoic acid (DHB) matrix and applied to a MALDI sample template. Mass spectra were recorded in Reflectron mode with delayed extraction ‘on’. Two hundred and fifty-six laser shots constituted one spectrum. Reanalysis for MS/MS confirmation of the N termini was performed with a 4700 Proteomics Analyser (Applied Biosystems).

For the digestion of spots, the gel pieces were destained in 500 μl destaining buffer [200 mM NH_4_HCO_3_, 50 % acetonitrile (ACN)], equilibrated in 500 μl digestion buffer (50 mM NH_4_HCO_3_, 5 % ACN). The supernatant was removed and the gel piece was dried within 30 min in a Microconcentrator 5301 (Eppendorf) at 30 °C, and then digested in 25 μl digestion buffer containing 0.1 μg trypsin (sequencing-grade modified trypsin, Promega). After digestion overnight at 37 °C, the reaction tube was centrifuged and the supernatant (S1) transferred into another tube. To the gel piece, 25 μl 60 % ACN, 0.3 % trifluoroacetic acid (TFA) was added to stop the trypsin reaction and to shrink and wash the gel piece. After 10 min the supernatant (S2) was added to S1. The gel piece was washed and shrunk with 25 μl 100 % ACN. This supernatant was then added to S1+S2 and dried in the Microconcentrator 5301 at 65 °C. For the MS analysis, the dried peptides were dissolved in 1 μl 33 % ACN and 0.1 % TFA, and 0.25 μl of this solution was mixed with 0.5 μl alpha-cyano-4-hydroxycinnamic acid (CHCA) solubilized in 50 % ACN, 0.3 % TFA on Parafilm, and the resulting mixture was transferred to the template of the mass spectrometer and analysed.

The peptide masses were obtained using the following parameters: reflectron mode, 20 kV accelerating voltage, a low mass gate of 500 Da, and a mass range between 500 and 4000 Da. MS/MS spectra were obtained with and without collision gas. The database searches were performed with Mascot (http://www.matrixscience.com). Search parameters were: 30 p.p.m. peptide mass tolerance for peptide mass fingerprints, and 0.3 Da for MS/MS spectra. MS/MS data were compared with the theoretical sequence data of the protein under investigation. Search criteria were: one and two missed cleavages allowed and possible oxidation of methionine; N-terminal acetylation of the protein; and pyroglutamic acid formation from N-terminal glutamine, propionamide and sodium adducts. MS/MS spectra were manually evaluated.

The MS/MS peaklists can be found within the 2D-PAGE database (http://www.mpiib-berlin.mpg.de/2D-PAGE/), linked to the relevant protein.

## RESULTS

### Confirming the *M. tuberculosis* GlpX start

Our approach arose from an earlier set of experiments in which we predicted that the annotated translational starts for two *M. tuberculosis* proteins were incorrect (Movahedzadeh *et al.*, 2004). This is despite the fact that this was one of the earliest completed genome sequences in which each ORF was manually annotated (Cole *et al.*, 1998), and which has since been rigorously reannotated (Camus *et al.*, 2002).

Our predicted reassignment of the start of GlpX (Rv1099c) was based mainly on extensive comparative genomic analysis (Movahedzadeh *et al.*, 2004). We observed that amino acid homology extended beyond the annotated start, and we proposed that translation actually started 34 residues earlier (Fig. 1). Crucially, some experimental N-terminal sequence data had been reported for the *Corynebacterium glutamicum* GlpX orthologue, Fbp (Rittmann *et al.*, 2003), which allowed us to be more confident about our new prediction for the *M. tuberculosis* GlpX. The extension of GlpX led to a conflict with the adjacent and divergently transcribed gene *Rv1100*, and we proposed an alternative start for this as well. This apparent misannotation therefore completely altered the predicted shared promoter region for two genes.

**Fig. 2. f2:**
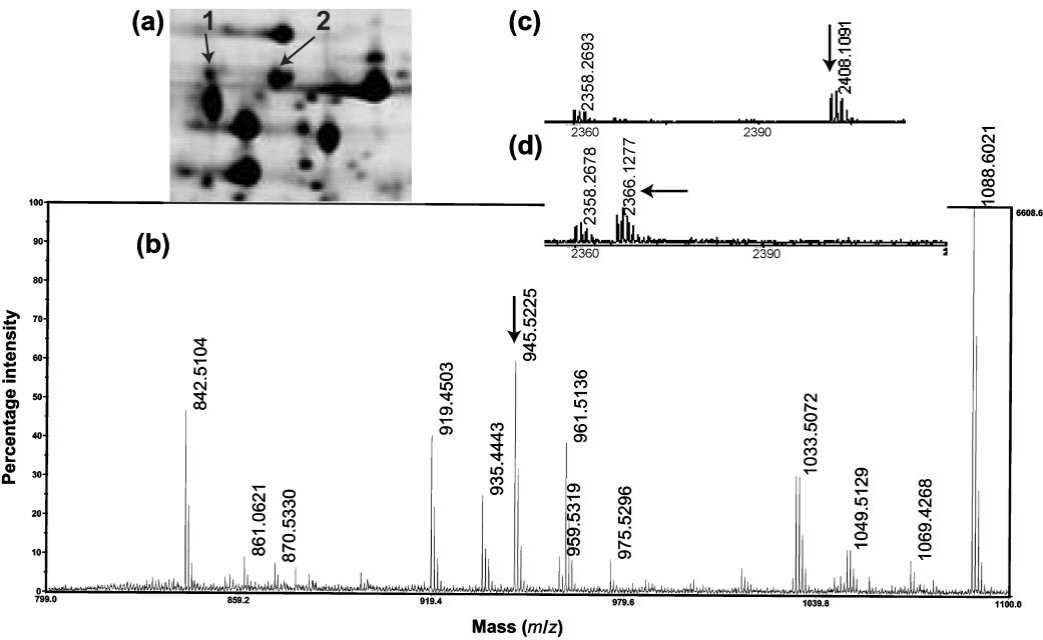
MS of GlpX. (a) SDS-PAGE indicating two forms of GlpX, acetylated (1) and non-acetylated (2). (b) Identification of a peptide spanning the annotated start (NLAMELVR=945.52; MS/MS-detected b ions: b2, b3, b4, b5, b6; detected y ions: y1, y2, y3, y5. (c) Identification of an acetylated N-terminal peptide (Ac-TAEGSGSSTAAVASHDPSHTRPSR=2408.1; MS/MS: y4, y5, y8, y9, y10, y21). (d) Identification of a non-acetylated N-terminal peptide (TAEGSGSSTAAVASHDPSHTRPSR=2366.1; MS/MS: y1, y4, y8, y9, y21).

As GlpX had been identified proteomically in *M. tuberculosis* extracts (http://web.mpiib-berlin.mpg.de/cgi-bin/pdbs/2d-page/extern/index.cgi), we reasoned that existing MS data might be able to confirm or refute our reannotation. We therefore predicted peptide masses that would distinguish the predictions (Fig. 1, Table 1). Analysis of the existing data was inconclusive, but generation of new MS data confirmed (a) the presence of a peptide (NLAMELVR) that crossed the originally predicted start site and (b) the presence of a peptide that corresponded to our new prediction (Fig. 2). No peak corresponding to the original start prediction was seen.

The start peptide that we identified (TAEGSGSSTAAVASHDPSHTRPSR) lacked an N-terminal formyl-methionine (fMet), which is not an unusual finding (see Discussion). Interestingly, GlpX occurred in two spots on the 2D gel; the more acidic one showed a shift of 42 in mass for this peptide, suggesting an acetylation (Fig. 2). MS/MS spectra resulted in y ions up to y21 without this shift, suggesting acetylation of the N-terminal threonine. We concluded that the start of the new prediction was correct, with posttranslational modifications to cleave fMet and partially acetylate the N terminus.

### Developing a higher-throughput approach

The success with GlpX showed the potential of peptide mass mapping to experimentally verify protein starts on a large scale. We therefore calculated the masses of start peptides for all predicted proteins in the *M. tuberculosis* genome. We called the currently annotated start codon ‘p0’, and identification of tryptic peptides starting at this codon would support the current annotation.

We then identified potential alternative start codons upstream (m1, m2, etc. for ‘minus’) and downstream (p1, p2, etc. for ‘plus’) (see Fig. 1); for each of these we recalculated the predicted masses of start peptides. Use of an upstream start codon would result in a longer protein, with the p0 codon-encoded residue contained within another peptide, the identification of which would be evidence against p0 (as occurred with GlpX). We therefore also calculated the masses of such trans-p0 peptides. The algorithm used is described in Methods, and shown in Fig. 1.

To test our approach more rigorously, a shortlist of 15 proteins was drawn up. This was based on (a) the presence of existing MS data, (b) no obvious signal sequence being present, (c) the mass of the p0 peptide falling within the range of ideal sensitivity for MS, and (d) the peptide being preferably cleaved by trypsin at arginine rather than lysine residues (see Methods).

Table 1 shows the data that we obtained. We initially analysed existing data, which were adequate in some instances but not in others, requiring repeat MALDI-MS analyses. Of the 15 proteins, we had difficulties with two. In one case (Rv1017c; PrsA), the predicted N-terminal peptide coincided with a common contamination peak. Another (Rv2557) came from a spot containing more than one protein and was therefore unusable for the present investigation. The other 13 proteins were successfully analysed, and are briefly presented below.

### Peptide start analyses

#### Confirmed starts.

We confirmed the starts for 11 proteins (see Table 1). In most cases, a peptide lacking fMet was detected, although in one case (Rv0738), deformylated methionine was present. In two cases (ArgD and PrcA), both N-terminally acetylated peptides were found in addition to the non-acetylated form.

#### Revised starts

##### GlpX (Rv1099c): m2.

As described above, our hypothesis that GlpX is 34 aa residues larger than originally predicted was confirmed (Fig. 1). The fMet is cleaved and, in addition to an unmodified protein species, there is also a less-abundant spot with an acetylated N-terminal peptide (Fig. 2).

##### RibH (Rv1416): m1.

**Fig. 3. f3:**
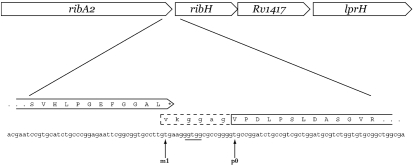
TSS reassignment for *ribH* (*Rv1416*). There is a 14 bp gap between the stop codon of *ribA2* and the *M. tuberculosis* annotation predicted start codon (p0) of *ribH*. No peak corresponding to this start was seen; however, peaks corresponding to the m1 start variant (dashed box) were identified. Thus, the corrected TSS overlaps the upstream *ribA2* (*Rv1415*) gene by 4 bp, an arrangement often observed in prokaryotic genomes for functionally related genes in a common operon.

The *ribH* gene lies downstream of *ribA2* in what appears to be an operon (Fig. 3). There is a 14 bp gap between the stop codon of *ribA2* and the predicted start valine of *ribH*. No peak corresponding to this start was seen; instead, there was a clear peak corresponding to the peptide crossing the originally predicted start. The m1 start lies 2 aa upstream of the peptide identified, and as the second residue is a lysine, should be cleaved in the procedure used. We identified both the predicted cleaved peptide (GGA…) and a peptide in which the trypsin had not cleaved (MKGGA…). Where there are alternative start codons (GTG, TTG), fMet is inserted rather than the usual amino acid that these would code for (Laursen *et al.*, 2005). The finding therefore of a methionine encoded by a GTG, which would normally code for valine, is in itself proof that this is the translational start. This sequence was confirmed by MS/MS. The peptide starting with GGA was confirmed by eight y ions (1, 2, 3, 5, 6, 9, 10, 12) and three b ions (4, 5, 8) and the peptide starting with MKGGA by eight y ions (1, 3, 4, 5, 6, 9, 10, 12). The expected ions y5 and y10, both with a cleavage after D and a mass loss of 64, further confirmed the sequence. This new *ribH* start would overlap with the *ribA2* stop codon (GTGA; Fig. 3) at position −4. Such an arrangement is known to be the most common for prokaryotic genes thought to be part of the same operon (Salgado *et al.*, 2000), as is the case with *ribA2* and *ribH*.

## DISCUSSION

We have shown that proteomic methods can be used to identify protein TSSs. Of 15 proteins tested, we confirmed predicted starts for 11, and reassigned the starts of two. In one case (*glpX*; *Rv1099c*), the reassigned start affects the adjacent gene and completely alters the predicted promoter region for both genes. In the second case (*ribH*; *Rv1416*), the alteration is minor, but makes biological sense, and indicates that a predicted ribosome-binding site is probably non-functional. As the *glpX* case was preselected, one of 12 randomly chosen proteins was misassigned. The sample is small, but indicates that a large number of proteins may be incorrectly annotated. While the correction of misassigned starts underlines the need to carry out this work, confirmation of current predictions is also an important contribution. As proteins are mainly identified through homology, it is critical that at least a small number of representatives have confirmed TSSs, in order to anchor the remaining predictions within an experimental context.

Our approach is simple in concept, and has the great advantage that most of the data are routinely generated during standard proteomic procedures. TSS identification has previously been a relatively neglected area; in the past, N-terminal sequencing was the main method for protein identification, but this has been largely supplanted by more sensitive MALDI-MS technology. There have been large-scale N-terminal sequencing projects; for example, Link *et al.* (1997) studied the N termini of 295 *E. coli* proteins. Of these, 72 failed because of signal peptide cleavage or N-terminal blockage. Of 223 N-terminal starts characterized, 10 (4.5 %) were reassigned. However, they used Edman degradation, and the need to use different chemistries makes this less practical as a routine method. We suggest that application of the method described here in proteomics laboratories would dramatically increase the number of experimentally determined protein TSSs, with minimal extra resources. There is also the possibility of retrospective examination using pre-existing MS datasets, although we preferred to carry out confirmatory analyses. This emphasizes the importance of storing MS data in proteome databases.

It would be possible to identify some of the predicted N-terminal peptides using a standard Mascot search, and the fact that this does not happen, or that the data do not feed into the wider community, may reflect different priorities (protein identification being the normal aim). However, our method goes further, and evidence for a start peptide is supported (a) by the absence of alternatives and (b) by the search for peptides spanning potential starts. We accept that as with all peptide mass mapping, while the identification of a single peak with a particular molecular mass provides supporting evidence for the presence of a particular peptide, additional MS/MS analysis to confirm peptide identity is required to be confident about the assignment.

More recent large-scale proteome projects have used proteomics to help genome annotation (Jaffe *et al.*, 2004; Lipton *et al.*, 2002). In a reanalysis of the *Mycoplasma pneumoniae* genome, over 81 % of the predicted ORFs were identified proteomically, and 16 new ORFs were detected. The algorithm used resulted in the extension of the predicted N termini of 19 proteins, on the basis that peptides were identified that extended beyond the annotated start. The translational start was then reassigned by looking upstream for a start codon. However, these groups did not actually identify start peptides.

We looked for proteins starting at ATG, GTG or TTG codons. ATG is the classical initiation codon, but in GC-rich genomes such as that of *M. tuberculosis*, GTG is extremely common. Our method conclusively confirms the starts in which a methionine residue is seen with a GTG or TTG codon, as this is the only scenario in which such a substitution would take place. Thus, methionine residues were detected with both RibH and Rv0738, even though they start with GTG (valine) codons.

There are limitations to our method. TSSs of proteins that are cleaved (for example removal of signal sequences) will not be resolved. However, it would be possible to adapt our method to identify such cleaved N termini, as signal sequence cleavage sites can be predicted. We deliberately chose a subset of proteins that would maximize the chance of success, suggesting that our success rate (84 %) would be less likely with a random set of proteins. One criterion we used was to choose proteins for which the predicted N-terminal peptide was readily resolvable using our experimental conditions and mass spectrometer. If a protein had an N-terminal peptide that was not suitable, then alternative proteases could be used, or an orthologue in another species might be more amenable. Also, the presence or absence of a peptide that crosses the predicted start might be detectable, even if the start peptide itself was not. We encountered another problem with Rv2908c, in which a predicted start (p2) corresponded to a tryptic peptide (because it lay next to an arginine or lysine residue); in such cases it would be necessary to include additional evidence before confidently assigning this as a start. Finally, our approach is directly applicable to all prokaryote genomes, but will need adapting for eukaryotes, in which exons need to be considered.

The applied method described here can use data from 2-DE/MS and LC/MS identifications. 2-DE/MS data have the advantage of high sequence coverage and therefore have a better chance to have access to the N terminus. The mass analysis of a 2-DE spot with peptide mass fingerprinting investigates only one protein, because 2-DE separates proteins. Therefore, the N-terminal peptide can be searched in a relatively small dataset of the proteolytically obtained peptides of this single protein. There is also access to MS and MS/MS data in already existing proteome databases (e.g. http://www.mpiib-berlin.mpg.de/2D-PAGE/). LC/MS methods have an advantage for sensitive identification of proteins, because only two or three peptides are sufficient for identification. This advantage turns into a limitation for the problem of the identification of posttranslational modifications and N termini (Schmidt *et al.*, 2004), because the probability that the N terminus or the modification is within the two or three identified peptides is very low. Nevertheless, our strategy for N-terminus confirmation or correction has the potential to be applied to the LC/MS approach by changing the experimental design to focus on the N-terminal peptide, as for example by the Cofradic approach (Gevaert *et al.*, 2003).

Both of the misannotated proteins that we identified were longer than the predicted proteins. Although our sample was small, we might expect this to be the case with a carefully annotated genome, as alignments to other homologues will tend to give a conservative indication of length. With genomes for which gene start assignment uses other assumptions, the balance of ‘m’ and ‘p’ reassignments might change.

One benefit of proteomic analyses is the ability to identify posttranslational modifications. A common modification is removal of fMet, and we observed this for 11 of the 13 proteins. In the two cases in which there was no cleavage, the residue had been deformylated. In some cases, methionine residues (both N-terminal and internal) had been oxidized (Schmidt *et al.*, 2006); we cannot tell if this occurred intracellularly or during the experimental procedure. Most interestingly, N-terminal peptides from three proteins (GlpX, ArgD and PrcA) were at least partially acetylated. MS/MS sequencing showed the acetyl group to be on the N-terminal residue; although N-terminal acetylation is the norm in eukaryotic proteins, it is reported to be rare in prokaryotes (Polevoda & Sherman, 2002). It is amino acid dependent (Persson *et al.*, 1985), and our observations of acetylated serine or threonine residues are in line with previous work. N-terminal acetylation may not always alter function, but in some cases it does; thus, the important *M. tuberculosis* antigen ESAT-6, which normally interacts with the protein CFP-10, fails to do so when acetylated (Okkels *et al.*, 2004). We show here that three out of 13 proteins (23 %) have an acetylated form. This raises the question of whether the assumption that few prokaryotic proteins are acetylated is true for all bacteria. Much of the existing dogma has come from the analysis of *E. coli* proteins, of which in a survey of 810 proteins (Polevoda & Sherman, 2003), only three were acetylated, all three being ribosomal subunits (S5, S18 and L12); our data suggest that the mycobacteria, at least, may be very different. A larger survey of proteins from these and other bacteria is needed to confirm this.

In this paper we suggest a simple strategy for the experimental identification of TSSs. The correct identification of these sites affects not only the length and possibly the function of the encoded proteins, but also key regulatory factors, such as the presence or absence of upstream regulatory sequences and the identification of genes likely to be co-expressed as part of an operon. We believe that the proposed nomenclature used herein for describing alternative start sites could be a useful standard for TSS identification in genome annotation. Since the data are often available and the strategy is therefore not particularly onerous in time or materials, we would recommend that researchers working on a particular gene attempt to mine proteomics databases for TSS identification, and that researchers performing large-scale proteomics investigations consider making TSS identification part of their protein-characterization procedure. The experimental identification of the TSS of a protein should be of use in annotating orthologues in other species, so that work in different laboratories would support a general effort to improve ORF annotation.

## Figures and Tables

**Table 1. t1:** MS analyses of N-terminal peptides from *M. tuberculosis* proteins MetOx, oxidized methionine.

**Gene name**	**Rv no.**	Spot no*	**Location†**	**Mass of N-terminal peptide**	**Sequence of N-terminal peptide‡**	**Prediction§**	**Comments**
*Rv0250c*	*Rv0250c*	5_19	CP	1781.93	(L)STTAELAELHDLVGGLR	p0	fMet cleaved
*Rv0313*	*Rv0313*	5_18	CP	1733.68	(V)GDYGPFGFDPDEFDR	p0	fMet cleaved
*rpmC*	*Rv0709*	5_154	CP	984.544	(M)AVGVSPGELR	p0	fMet cleaved
*Rv0738*	*Rv0738*	3_350	CP	1130.51 (2 MetOx)	[V]MoxDPLMoxAHQR||	p0	MS/MS confirmed. 2MetOx detected; thus fMet deformylated but not cleaved. GTG start codon
*greA*	*Rv1080c*	5_65	CP	1816.74	(M)TDTQVTWLTQESHDR	p0	fMet cleaved
*glpX*	*Rv1099c*	1_249/1_216	CP	2408.1	(M) Ac-TAEGSGSSTAAVASHDPSHTRPSR¶	Ac-m2/m2	MS/MS confirmed. fMet cleaved. Both acetylated and non-acetylated forms found
*Rv1109c*	*Rv1109c*	3_443#	CP	834.62	(M)ATAPYGVR	p0	fMet cleaved
*ribH*	*Rv1416*	5_97	CP	1842.94 (MetOx)	[V]MoxKGGAGVPDLPSLDASGVR||	m1	MS/MS confirmed. fMet deformylated but not cleaved. GTG start codon
*Rv1558*	*Rv1558*	6_33	CP/CSN	1674.79	(M)PLSGEYAPSPLDWSR	p0	fMet cleaved
*argD*	*Rv1655*	1_386/2_17	CP	1155.52 (MetOx)	(M) Ac-TGASTTTATMoxR**	Ac-p0/p0	fMet cleaved. Both acetylated and non-acetylated forms found. Internal Met oxidized
*prcA*	*Rv2109c*	3_325/3_372	CSN	1630.74 (MetOx)	(V) Ac-SFPYFISPEQAMoxR††	Ac-p0/p0	fMet cleaved
*Rv2908c*	*Rv2908c*	6_19‡‡	CP	1393.76	(M)SAVVVDAVEHLVR	p0	fMet cleaved
*Rv3143*	*Rv3143*	5_149	CP	846.42	(V)PDSSTALR	p0	fMet cleaved

*All from *M. tuberculosis* unless otherwise mentioned. †CP, cellular protein; CSN, culture supernatant. ‡Parentheses indicate a missing residue, presumed to be a cleaved fMet, and the amino acid predicted from the DNA sequence. §Identity of start peptide. p0 indicates that the annotation in the published genome sequence is correct. m1/m2 indicates that the first or second upstream start codon is used. ‘Ac’ indicates that the peptide is N-terminally acetylated. ||Brackets indicate the codon present where an uncleaved methionine was identified (M=ATG, V=GTG, L=TTG). ¶Non-acetylated peptide also found, mass 2366.1. #Spot from *Mycobacterium bovis* BCG Chicago. **Non-acetylated peptide also found, mass 1113.52. ††Non-acetylated peptide also found, mass 1588.74. ‡‡Spot from *M. tuberculosis* Erdman.

## Abbreviations

- 2-DE, 2D gel electrophoresis
- fMet, formyl-methionine
- TSS, translational start site
